# Will Smart Improvements to Child Restraints Increase Their Popularity?

**DOI:** 10.3390/ijerph192315727

**Published:** 2022-11-25

**Authors:** Li Jiang, Mei Zhao, Hao Lin, Haiyuan Xu, Xiaojiao Chen, Jing Xu

**Affiliations:** 1 CAS Key Laboratory of Mental Health, Institute of Psychology, Chinese Academy of Sciences, Beijing 100101, China; 2 Department of Psychology, University of Chinese Academy of Sciences, Beijing 100049, China; 3 Shanghai Woyoo Electronic Technology Co., Ltd., Shanghai 201112, China

**Keywords:** child restraints, Isofix, temperature sensing, forgotten alarm, voice control, acceptance, purchase intention

## Abstract

In developing countries, child safety seat use remains low, which contributes to the consistently high rate of child injuries and deaths in traffic accidents. In order to protect the safety of child passengers, it is necessary to improve the public acceptance of child restraints. We improved the shortcomings of the traditional child restraints by adding some new features: 1, tightening Isofix automatically; 2, using temperature sensing, a high-temperature alarm, automatic ventilation, and cooling; 3, using pressure sensing, if the child is left alone it will set off the car alarm; 4, voice control to adjust the angle of the backrest; 5, the seat can be folded into the trunk. These functions make human-computer interaction more humane. The authors collected changes in parental acceptance of child restraints using the interview method and questionnaires. We found that acceptance increased significantly after making intelligent improvements to the child restraints. The authors used the Technology Acceptance Model to identify the key caveats influencing users’ use of intelligent child restraints. Performance expectations, effort expectations, social influence, convenience, and hedonic motivation positively and significantly impacted the willingness to use intelligent child restraints, so the authors suggest that these points should be emphasized when promoting the product. The current study findings have theoretical and practical implications for smart child restraint designers, manufacturers, sellers, and government agencies. To better understand and promote child restraint, researchers and marketers can analyze how people accept child restraint based on our research model.

## 1. Introduction

It is well known that the use of child restraints has reached more than 80 percent in developed countries, such as the Commonwealth countries and the majority of European Union nations, which have laws mandating the use of child restraints [1]. In developing countries, however, the usage rate is low because many countries lack legislation regarding the use of child restraints [1,2]. For instance, the usage rate of child restraints in India is only 5% [3], and the actual usage rate of CRS (Child Restraint System) for children under six years old in China, the world’s largest producer of child restraints, is only 17.3% [4]. To prevent children from being injured in traffic accidents, it is necessary to increase the rate of child restraint usage [1]. Although China has not yet enacted a national mandatory child restraint law, some provincial or municipal regulations are in place: Shanghai (March 2014), Shandong Province (August 2014), Shenzhen City (January 2015), Nanjing (May 2016), and Hangzhou (beginning 1 June 2016) [5]. Although the child restraints usage rate in these locations has increased since the introduction of regulations, with the actual usage rate of child restraints for children aged 0–3 years reaching 30% in Shanghai [6] and 22.9% in Shenzhen [7], it is still far below expectations; consequently, regulations alone cannot rapidly increase the child restraints usage rate in China, and researchers want to increase the use of child car seats in developing countries by researching and improving the experience of using CRS and adding new functions.

In 2016, we collected the views and opinions of young Chinese parents on child restraints through interviews and focus groups, and found that they were reluctant to use child restraints for the following reasons: (1) the child restraints are too large, the bucket-shaped seat structure will make the child feel very repressed, generating strong opposition; (2) they occupy space and are inconvenient to store; (3) the installation is too challenging; (4) lack of comfort. Recent studies have expressed similar opinions [8]. To increase the acceptance of child restraints, after two years of research and development, we designed and manufactured a smart seat that addresses the deficiencies of conventional child restraints and adds the following new features: (1) an electric Isofix tightening, and automatic installation of the seat; (2) increased temperature sensing, high-temperature alarm in the car, automatic ventilation, and cooling; (3) increased pressure sensing, and alarm when the child is left alone in the car; (4) voice control to adjust the angle of the backrest, thereby enhancing the ride’s comfort; (5) the seat can be folded and stored within the trunk. These features satisfy customer needs in full and make human-computer interaction more humane.

Every year, there are reports of children who die from heat-related injuries after being left in cars by their parents. Wen-Song Zhao et al., who are associate researchers at the Road Traffic Safety Research Center of the Ministry of Public Security, found in 2017 that the majority of accidents involving children left in cars occurred between May and July, accounting for 80% of the cases, and may be closely related to the high summer sun exposure of vehicles. The vast majority of statistical accident cases involved children aged three to five, who accounted for 81% of the total [9]. The new feature on smart child restraints that alerts parents when they leave their kids alone in the car and triggers the alarm for high temperatures in the car can help stop these kinds of accidents.

In the previous literature, the focus has been on studies that use age and size-appropriate child safety seats to prevent child crash injuries and deaths [10,11,12,13], pediatric research on child safety seats [14,15,16,17,18] and the prevalence of their use in specific areas [19,20].

Previous studies on the functional improvement of child safety seats have remained in the experimental model stage [10,11,12,13] and have not been commercialized into products for sale to the general public, so people do not have access to child restraints with new features. Our research has transitioned from the laboratory to the marketplace. It is now sold in shopping malls in Beijing and Shanghai, China, as well as at Shanghai Bentley automobile sales service shops in Shanghai. However, these three years of sales have not met expectations, which raises the following questions.

(1) Why are sales of these new smart child restraints so low, even though we have fixed the problems with traditional child restraints to meet customer needs?
(2) What must be done to increase the impact of intelligent child restraints on the public?

### Theoretical Context

This intelligent child restraint is very different from the conventional child restraint in that it has a battery and can be operated with a mobile app via Bluetooth. As a result, it falls under the category of new technology.

Based on the compilation of essential research on the technology acceptance model (TAM) carried out in recent years, the unified theory of acceptance and use of technology (UTAUT) was presented by Viswanath et al. [21].

UTAUT2 integrates the hedonic incentive structure, the price value structure, and the habit structure with UTAUT. It is expected that individual variations (e.g., age, gender, and experience) will modulate the influence of these structures on the intention to use [22].

There are studies in the prior literature that apply the UTAUT2 (Unified Theory of Acceptance and Use of Technology) model [22] to research areas such as telemedicine [23,24], automated vehicles [25,26,27], wearable fitness and health technology or smart homes [28], smartphones [29], online banking [30], mobile health [28] and other personal application fields. However, no studies apply this theory to the behavioral intention of using an intelligent child restraint. Due to this, we took UTAUT2 as our starting point and made some minor modifications to make it more applicable to the field of intelligent child restraint. A technology acceptance model evaluates its acceptance and the various factors influencing it. As a result, the following were the study’s main goals:

(1) To find out if intelligent child restraints are more popular with parents than traditional child restraints.
(2) What makes people more likely to buy smart child restraints, and what makes them less likely to do so?
(3) What needs to be done to get more parents to use smart child restraints so that more kids do not get hurt in car accidents?

## 2. Materials and Methods

### 2.1. The Introduction of Intelligent Child Restraints

**Figure 1:**
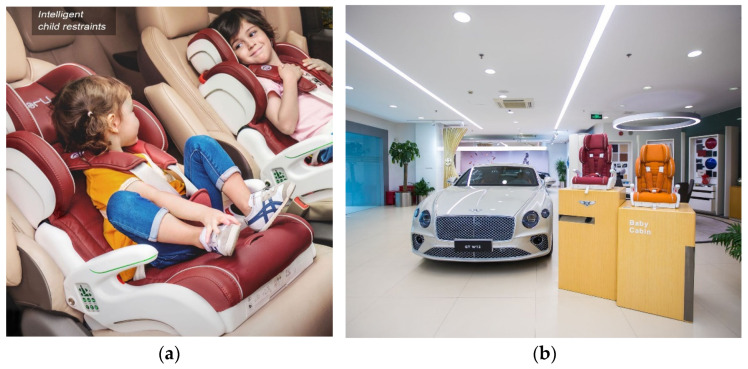
(**a**) Intelligent child restraints appearance; (**b**) Intelligent child restraints are sold in Shanghai Bentley automobile sales service shop.

This product is suitable for children weighing 9 kg to 25 kg (about nine months to six years old). Figure 1 shows the intelligent child restraints appearance, which is available in vehicle dealerships in China.

#### 2.1.1. One-Touch Isofix Installation

**Figure 2:**
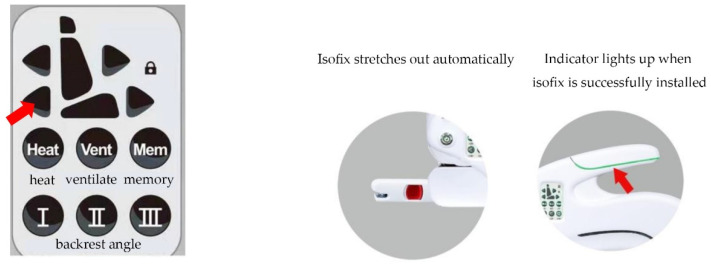
Control Isofix automatic extension with the control panel on the seat.

Double-click the Isofix out button on the seat control panel, and the Isofix will be stretched automatically. Push the seat to install the Isofix, and the indicator light will light up when the Isofix snaps into place. Figure 2 shows the Isofix control keys and the electric Isofix.

#### 2.1.2. The 120-Degree Backrest Has Infinite Adjustment and Three Memory Positions

**Figure 3:**
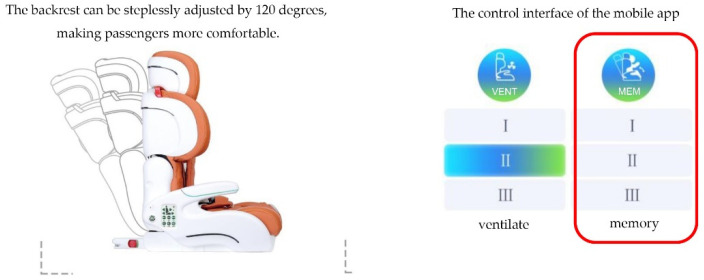
The 120-degree backrest has infinite adjustment and three memory positions.

The seat control panel, a cell phone app, and voice commands can all be used to change the angle of the backrest and save three settings. Figure 3 shows the three angles of the seat backrest that can be stored via memory keys.

#### 2.1.3. Heating and Ventilation Systems

**Figure 4:**
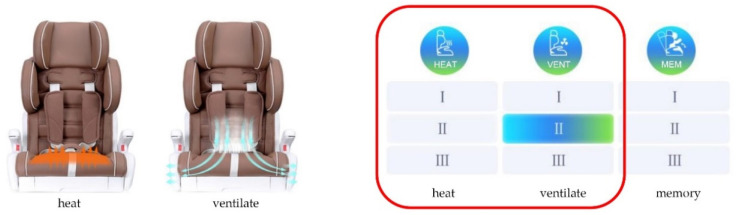
Heating and ventilation systems.

To make the seats more comfortable, they have heating and cooling features with three levels that can be controlled using the seat control panel, a mobile app, or your voice. Figure 4 shows the APP control interface of the intelligent child restraints.

#### 2.1.4. Automatic Tightening of the Backrest and Car Seat

**Figure 5:**
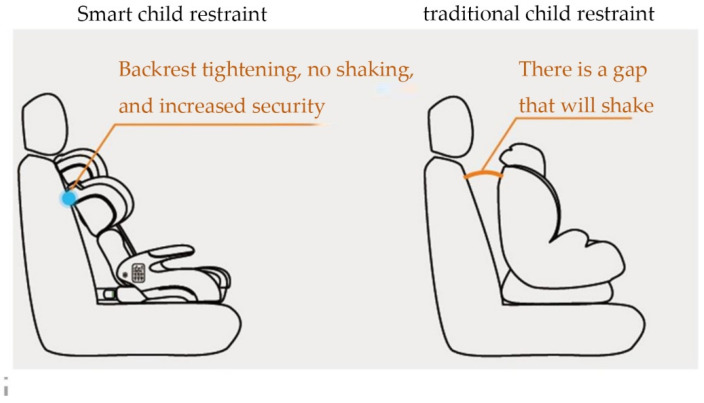
Comparison of intelligent safety seat backrest and traditional safety seat.

Every 60 s, it automatically detects whether the Isofix is locked. It does not require any operation. When traveling on rocky roads, it can be locked firmly in real-time. Figure 5 compares intelligent child restraint backrests and traditional child restraints.

#### 2.1.5. Out-of-Seat Safety Alert

**Figure 6:**
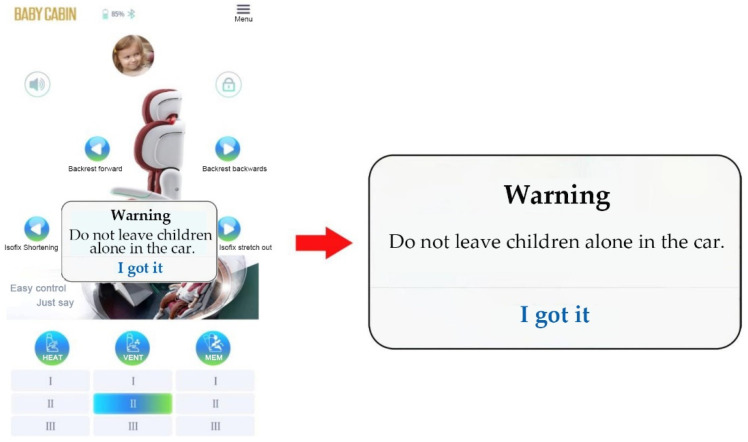
Out-of-seat safety alert.

Intelligent child restraints have an out-of-seat reminder function to prevent children from being left alone in the car. The mobile app automatically alerts the driver when an adult leaves the vehicle, and the pressure sensor detects that the child is still in the seat. Figure 6 shows the app alert message.

#### 2.1.6. Foldable

**Figure 7:**
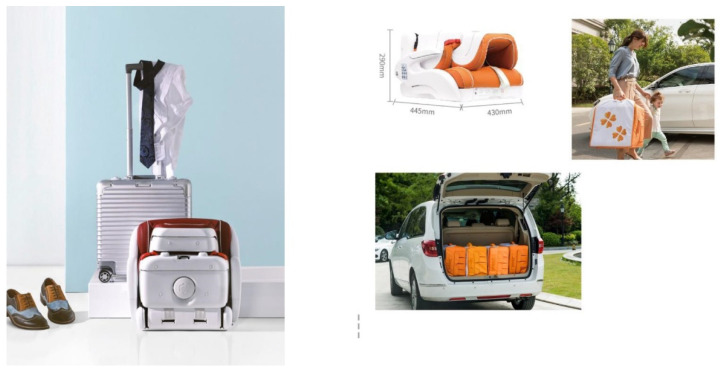
Foldable.

It can be folded into an 18-inch luggage size, so storage is convenient. Figure 7 shows the intelligent child restraints folding function and compares it with the 18" Luggage.

#### 2.1.7. App for intelligent child restraints

**Figure 8:**
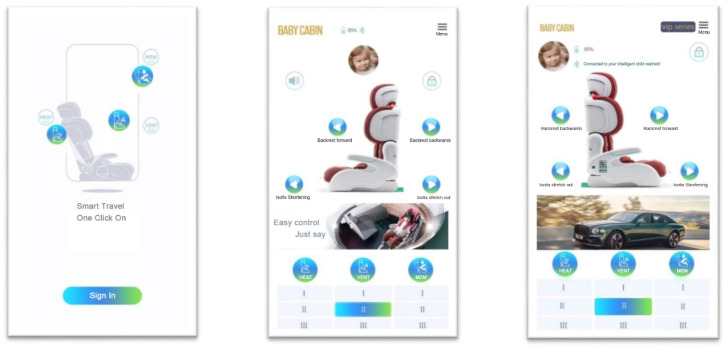
It can be controlled using a cell phone APP.

Download the mother and child intelligent control center app to your cell phone and connect it to the smart child restraints via Bluetooth to control it. Figure 8 shows the page of the APP.

#### 2.1.8. Voice Control System

**Figure 9:**
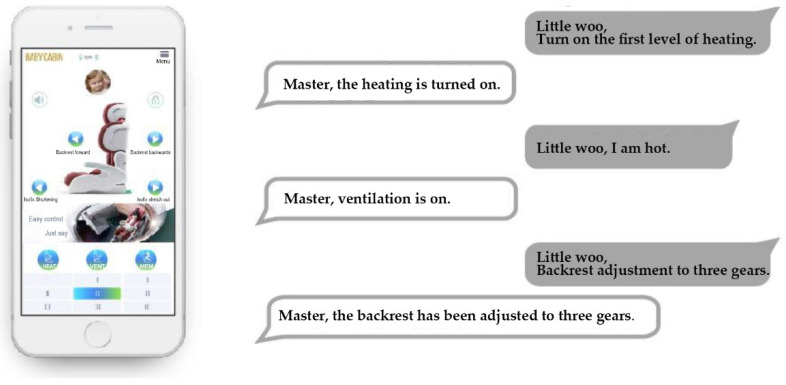
It can be controlled using speech.

Speaking is one way to control it. In Figure 9, the human-issued commands are displayed in the gray dialog box, and the intelligent restraint’s voice responses are displayed in the white dialog box.

#### 2.1.9. The Central Control Panel of Vehicles

**Figure 10:**
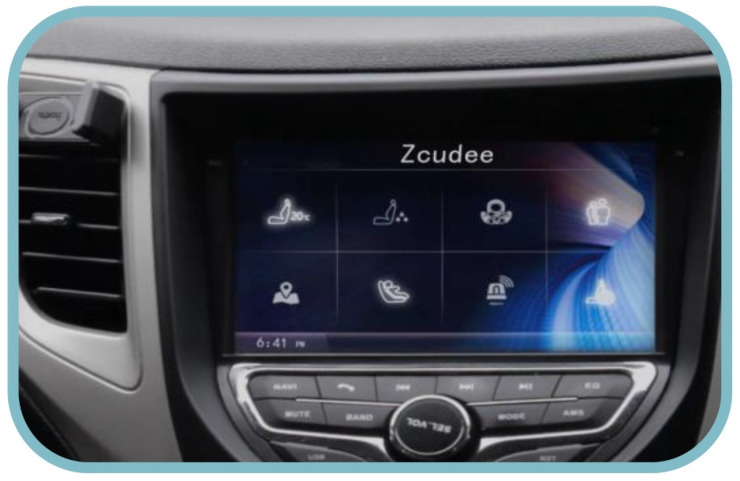
The central control panel of vehicles.

This intelligent child restraint can be connected to the car and controlled from the vehicle’s center console, making it safer to drive. Figure 10 depicts the vehicle’s central control panel’s user interface.

### 2.2. Participants

For this study, a total of 1107 respondents were recruited, all of whom came from homes with children ages 1–4, owned private vehicles, and held valid driver’s licenses.

### 2.3. Test Method

In August 2022, an invitation was sent to parents of small kindergarten classes in Shanghai, inviting them to try out the intelligent child restraints in Sogo Shanghai and Shanghai Bentley Automobile Sales Service Shop. Each parent filled out the questionnaire online after trying it for one hour. The link to the online questionnaire was displayed on three official websites: Questionnaire.com, Questionnaire Star, and Tencent Questionnaire. They could see the questionnaire by visiting the above three websites and voluntarily filling it out. We promise to keep the data confidential. 

The questionnaire consisted of three parts: Part 1: an introduction to the intelligent child seat via file, image, and video; Part 2: a questionnaire scale; and Part 3: demographic information about the subjects. The questionnaire used a seven-point Likert scale. As proposed by [31], all existing scales were changed and translated with the assistance of two bilingual academics to ensure the authenticity of the translation. The scale questions for the questionnaire are in Appendix A.

**Figure 11:**
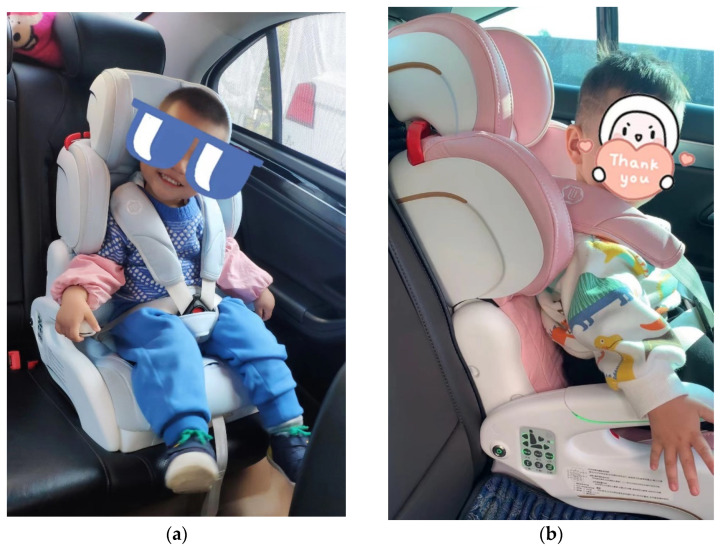
(**a**,**b**) The smart child restraints were installed in the participant’s vehicle.

As shown in Figure 11, the smart child car seats were installed in the participant vehicles.

### 2.4. Data Collecting and Filtering

To ensure the reliability and validity of the model, a pretest was conducted on 43 senior white-collar officers who graduated from the MBA2015 class of Tongji University in Shanghai on 24 April 2022.

In the end, 1107 surveys were collected. In two stages, data were filtered. Initial screening eliminated people with inconsistent sociodemographic information. To define the target population, those who said they did not own a car or had no children were excluded from the survey. 

After filtering complete questionnaires, 1020 valid questionnaires were obtained, including 389 males and 631 females. Table 1 shows the demographic information of the respondents. Most of the respondents said they have or will have a higher education degree, including an associate degree (18.2%, *n* = 186), a bachelor’s degree (63.2%, *n* = 645), and a master’s degree or higher (9.4%, *n* = 96). Approximately 46.4 percent of these respondents have an annual household income of 100,000 to 300,000 yuan, and approximately 23.8 percent have an annual household income of 300,000 to 500,000 yuan. The respondents’ ages ranged from 26 to 45 years old (26 to 35 years old, 57.1%, *n* =582; 36 to 45 years old, 21.0%, *n* =214). Table 1 outlines the basic situation of the respondents.

### 2.5. Hypothesis Development

**Figure 12:**
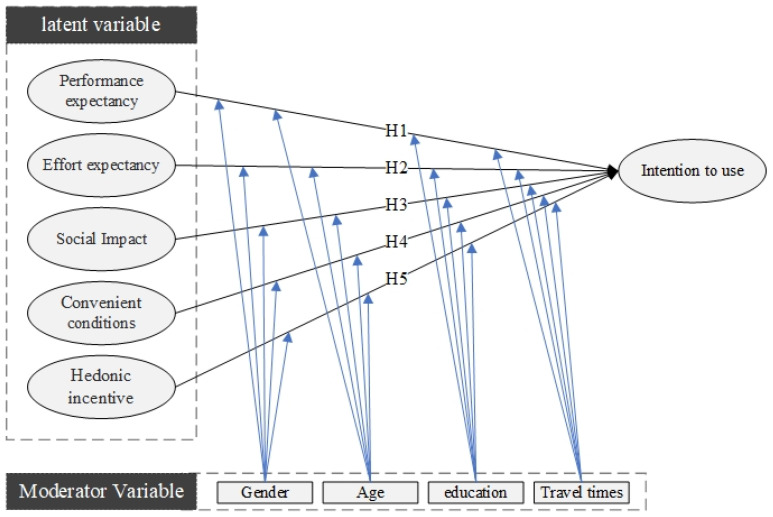
Research model.

This study presents the results of a questionnaire designed to analyze and simulate the acceptance of smart child car seats based on the UTAUT2 model structure. Following are the two sub-research aims of this study: Examine the impact of UTAUT2 on individuals’ behavioral intent to utilize smart child safety seats by establishing performance and effort expectations, social impact, facilitation circumstances, and hedonic incentive. Second, to investigate whether moderating variables (gender, age, level of education, and travel frequency) influence the interaction with these structures. The research model for this paper is depicted in Figure 12.

#### 2.5.1. Influences of Customer Performance Expectations on the Intention to Use Child Restraints

Performance expectation (PE) is the perceived beneficial influence of technology on users’ work [32]. It is also referred to as performance expectation in TAM/TAM2/TAM3 [21,33,34]; we also used the same terminology in our investigation, which is described as the degree to which a person thinks using a new product or technology enhances the performance of an application [32]. In addition, according to Venkatesh, several related constructs from past studies also contribute to usefulness [32]. In light of the preceding reasons, the following hypothesis is advanced:

**H1:**  *An essential and robust positive predictor of behavioral intention is an individual’s performance expectation*.

#### 2.5.2. Influences of Customer Effort Expectancy on the Intention to Use Child Restraints

A person’s “effort expectancy” (EE) is the amount of work they anticipate putting into a technological task [32]. In UTAUT/UTAUT2, effort expectation is taken into account as a potential explanatory variable for behavioral intention [22,32]. This element is also known as perceived ease of use in the TAM/TAM2/TAM3 models [32,33,35]. In this situation, the following hypothesis is derived:

**H2:**  *An essential and robust positive predictor of behavioral intention is an individual’s effort expectancy.*

#### 2.5.3. Influences of Customer Social Impact on the Intention to Use Child Restraints

When consumers utilize a product or service, they are nevertheless affected by the people and circumstances around them, a phenomenon known as “social impact” [32]. Social impact has been one of the significant determinants and a long-studied concept in information systems adoption research [22]. The consequently presented hypothesis is as follows:

**H3:**  *An essential and robust positive predictor of behavioral intention is an individual’s social impact.*

#### 2.5.4. Influences of Customer Convenient Conditions on the Intention to Use Child Restraints

A convenient condition is “consumers’ views of the resources and assistance available to accomplish a behavior” [22]. In light of the preceding reasons, the following hypothesis is advanced:

**H4:**  *An essential and robust positive predictor of behavioral intention is an individual’s convenient conditions.*

#### 2.5.5. Influences of Customer Hedonic Incentive on the Intention to Use Child Restraints

Because it added an emotional dimension to the previously primarily cognitive UTAUT, the hedonic incentive is often regarded as the most significant theoretical development in UTAUT2. One of the most influential factors in adopting and using new consumer technologies is the “hedonic incentive,” or the enjoyment one gets from interacting with the device in question [36,37,38,39]. It is from this that we infer the following hypothesis:

**H5:**  *An essential and robust positive predictor of behavioral intention is an individual’s hedonic incentive*.

### 2.6. Data Analysis

This study explored structural equation modeling with SPSS 25 and Amos24.0.0 to verify and assess the explanatory power of the modified UTAUT2 model.

The data were analyzed using a two-step approach [40]. 

In the first stage, the variable connections between latent variables and observable variables were evaluated using confirmatory factor analysis. The psychometric features of the measuring model were examined using indicator reliability, internal consistency reliability, convergent validity, and discriminant validity. Convergence validity was assessed using four criteria [40,41,42]:

(1) All factor loadings should be more than 0.50 on their respective scales.
(2) The extracted mean variance (AVE) should be more than 0.50.
(3) Construct reliability (CR) should be greater than 0.70.
(4) Cronbach’s alpha value should be greater than 070.

The discriminative validity of the data was tested according to the square correlation test of Anderson and Gerbing [40]. The correlation coefficient between the two latent variables should be less than the square root of each latent variable’s mean-variance extraction (AVE).

The fit indices used to determine how well the model fits the data were as follows:

χ^2^/df (ratio of chi-square to degrees of freedom) < 5, GFI (goodness-of-fit index) > 0.9, RMSEA (Root Mean Square Error of Approximation) < 0.10, RMR (root mean square residual) < 0.05, CFI (Comparative Fit Index) > 0.9, NFI (normed fit index) > 0.9, NNFI (non-normed fit index) > 0.9 [42].

The second step in the analysis was to study the structural model. The significance level and standardized regression weight of each potential variable relationship were studied. [43].

## 3. Results

### 3.1. Respondents’ Acceptance of Child Safety Seats Was Compared

As shown in Table 2, 28.31 percent of people in 2016 had an accepting attitude towards child restraints (the sum of scores 5, 6, and 7). In 2022, 61.87 percent of respondents would like to purchase a smart child restraint, 60.19 percent of respondents intend to use the smart child restraint extensively in the future, and 56.77 percent would like to try the smart child restraint. The acceptability of child safety seats in 2022 was higher than the acceptability of child safety seats surveyed in 2016.

### 3.2. The Price Respondents Are Willing to Pay

It can be seen from Table 3 that 31.96% of respondents can accept the price of a child safety seat below 3000 yuan, and 31.76% can accept the price between 3000 yuan and 4000 yuan. It can be seen that 63.72% of respondents can accept a price range below 4000 yuan.

### 3.3. Testing of Validity and Reliability

Table 4 shows the findings of the reliability analysis. Cronbach’s α of the six latent variables was all above 0.70, the factor loadings (*ƛ*) were higher than 0.5, the average variance extracted (AVE) was more than 0.50, and the composite reliability (CR) scores were also above 0.70, indicating that the questionnaire has excellent reliability [42].

As shown in Table 5, the KMO (Kaiser-Mayer-Olkin) value of the whole questionnaire was 0.984, which passed the Bartlett sphericity test and was significant. The factor loadings (ƛ) and average variance extracted (AVE) of each factor were greater than 0.5, indicating that the scale had good validity [41].

Discriminant validity requires that the correlation between two constructs be smaller than the square root of the average variance extracted For discriminant validity [40]. The measuring model demonstrated good discriminant validity, as shown in Table 6.

### 3.4. Structured Equation Modeling Results

χ^2^/df (ratio of chi-square to degrees of freedom) < 5, GFI (goodness-of-fit index) > 0.9, RMSEA (Root Mean Square Error of Approximation) < 0.10, RMR (root mean square residual) < 0.05, CFI (Comparative Fit Index) > 0.9, NFI (normed fit index) > 0.9, NNFI (non normed fit index) > 0.9 [42]. As can be seen from Table 7, the model has a good fit.

### 3.5. Path Hypothesis Test

**Figure 13:**
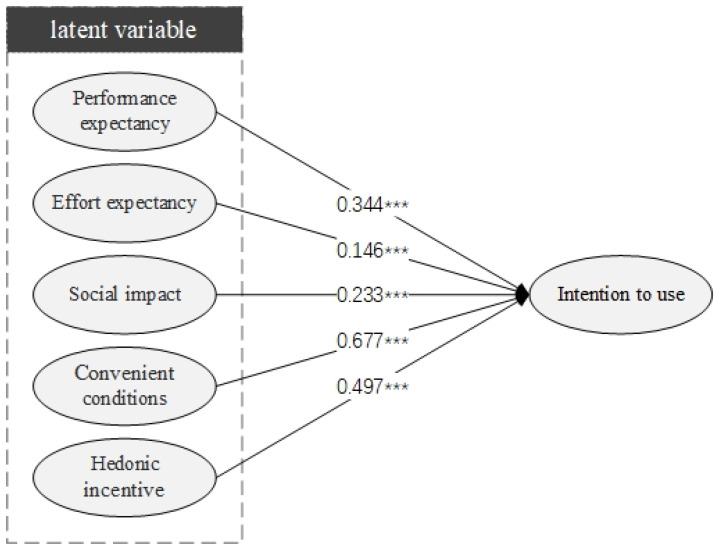
Findings from tests of hypotheses (Note: *** denote 1% significance levels).

The model path coefficient in Table 8 and Figure 13 shows that the significance *p* value of performance expectation → intention to use smart child safety seats was less than 0.001. Therefore, their relationship was significant, H1 was effective, and its influence coefficient was 0.344. For effort expectancy → willingness to use intelligent child safety seats, the significance *p* value was less than 0.001. So, their relationship was significant, H2 was valid, and its influence coefficient was 0.146. Social impact → intention to use smart child safety seats, the significance *p* value was less than 0.001. Therefore, their relationship was significant, H3 was valid, and its influence coefficient was 0.233. Convenient conditions → intention to use the smart child safety seat, the significance *p* value was less than 0.001. Therefore, their relationship was significant, H4 was valid, and its influence coefficient was 0.677. For hedonic motivation → intention to use smart child safety seats, the significance *p* value was less than 0.001. So, their relationship was significant, H5 was valid, and its influence coefficient was 0.497.

### 3.6. Invariance of the Multi-Group Structural Equational Model (MGSEM)

We examined the UTAUT2 model’s sensitivity to moderating factors such as gender, age, education level, and family vacation frequency. Byrne and Barbara proposed that a multi-group analysis could be used to categorize moderating variables [44]. Dummy variables are set to facilitate research: male (GEN = 0), female (GEN = 1); below 35 years old (AGE = 0), above 35 years old (AGE = 1); below a bachelor’s degree is a low degree (EDU = 0), a bachelor’s degree or above is a high degree (EDU = 1); less trips (TRA = 0, representing “less than 10 trips with children per month”) and more trips (TRA = 1, representing “11–20”, “21–30”) [45].

When comparing groups, the importance of measurement invariance cannot be overstated. Without measurement invariance, a between-group difference cannot be interpreted unambiguously. CFI less than or equal to –0.01 implies invariance should not be rejected [46].

Therefore, if the fit difference between a freely estimated and restricted model is slight (for instance, Rho = 0.05), then a modeling rationale may be adequate for deciding whether or not measurement equivalence is plausible [47]. These implicit heuristics should not be used as hard and fast rules but in the context of a researcher’s overall research aims, as every substantive application has particularities [48]. Table 9 shows the results calculated using Amos 24.

- Nested Model Comparisons for gender

There was no statistically significant difference between the two constrained models according to the χ^2^ (*p* = 0.286, *p* > 0.05), and ΔCFI < 0.01, ΔTLI < 0.05, indicating that factor loadings were invariant for both gender groups.

- Nested Model Comparisons for age

There was no statistically significant difference between the two constrained models according to the χ^2^ (*p* = 0.429, *p* > 0.05), and ΔCFI < 0.01, ΔTLI < 0.05, indicating that factor loadings were invariant for both age groups.

- Nested Model Comparisons for education

There was no statistically significant difference between the two constrained models according to the χ^2^ (*p* = 0. 0.704, *p* > 0.05), and ΔCFI < 0.01, ΔTLI < 0.05, indicating that factor loadings were invariant for both education groups.

- Nested Model Comparisons for travel times

There was no statistically significant difference between the two constrained models according to the χ^2^ (*p* = 0.013, *p* < 0.05), and ΔCFI < 0.01, ΔTLI < 0.05, indicating that factor loadings were invariant for both travel times groups.

## 4. Discussion

This study focusing on user needs uses the modified UTAUT2 model [22] to understand users’ acceptance of intelligent child restraints. It analyzes the main psychological components that affect users’ willingness to use intelligent child restraints. Although previous studies have proved that the use-rate of child restraints can be improved through legislation, in many developing countries the laws on the mandatory use of child restraints still need to be comprehensive and based on the best evidence. The American Academy of Pediatrics (AAP) guidelines for child safety seat legislation is based on the best evidence in the literature [49]. As shown in Table 2, public acceptance of intelligently modified child safety seats in 2022 was significantly greater than that of conventional child safety seats in 2016. Consequently, from the perspective of meeting users’ needs, the transformation of conventional child restraints may be a more effective and expedient strategy for increasing the utilization rate of child car seats.

The improved UTAUT2 model proposed in this study helps to extend its applicability in the child restraints industry. The empirical results show that the model fits well with the sample data, confirming the hypothesis path. In the structural model, performance expectation, effort expectation, social influence, convenience conditions, and hedonic incentive positively and significantly affect the intention of using child restraints after intelligent modification. Although there have been no studies on UTAUT2 in the child safety seat industry, this result is consistent with the UTAUT2 literature in other industries [25,27,28,29,30].

The convenience condition has the highest coefficient path weight among these five factors. That was probably because smart child restraints are very different from traditional child restraints: it is electric, for one thing, and there are buttons on the side of the seat that controls the Isofix retractable, backrest adjustment, and ventilation heating. Second, users must install an app on their phones to control it. The biggest obstacle to people accepting the improvements was the fear that the seats will not fit in their cars or that the app will not work with their phones. So, once users learned that smart child safety seats could fit into their car seats and that the app could be installed on both Apple and Android operating systems, they were significantly more likely to accept smart seats.

Hedonic motivation ranked second among all influencing factors, with a path coefficient of 0.497. The results demonstrated just what the designers intended. Six years ago, when the designers saw children crying in the traditional child safety seats, and the parents were helpless, they decided to adapt the traditional child safety seats to meet the customer need, i.e., to enhance the ride experience for children. Electric car seats make kids feel fun and comfortable, so they want to sit in them. Parents can also have more peace of mind behind the wheel. Therefore, users with high hedonic motivation are more willing to adopt intelligent child safety seats.

Performance expectation positively correlates with intending to use the intelligently modified child restraints, and this influencing factor ranks third. This result is consistent with other industry research results that performance expectations positively influence usage intention [29]. This result showed that if the child safety seat can meet the safety and functional needs of the user, then people will be more willing to use it.

Effort expectation was positively correlated with intelligent child restraints and had a minor effect among all factors. Effort expectation refers to how difficult it is for users to perceive their understanding and operation of intelligent safety seats when using them. Because the smart seat installation and removal are electric, it is much simpler than the traditional safety seat. In addition, these interviewees tried the experiment after watching the instruction video. They may have felt that the understanding and operation were relatively simple, so the effort expectation was not the most critical factor influencing their acceptance of smart child restraint.

Social influences also significantly impact children’s willingness to use the smart car safety seat. This result suggests that other peoples’ opinions influence the user’s behavior when using a safety seat. In today’s society, online shopping is becoming increasingly popular, and people are used to searching for others’ comments on the product online before they buy it. These comments on the Internet can significantly affect peoples’ purchase intentions. Therefore, the authors believe that the social influence here not only refers to the influence of friends and relatives but also includes the influence of other users’ comments on the Internet.

In addition, we explored the moderating effects of gender, age, education level, and child travel frequency on the relationships between UTAUT2 and a moderating structural model by creating performance and effort expectations, social influence, hedonic incentives, convenient conditions, and behavioral willingness. The results showed that the moderating variables have no significant effect on the relationship between the models. This result may be because both men and women, old and young, highly educated and poorly educated, frequent travelers and infrequent travelers, all want their children to be happy and quiet in the child safety seat. This result also indicates that the scale in this study can be applied to all users.

The results of this study verify the predictive value of the model. It is confirmed that if the modification of child safety seats can match users’ performance expectations, meet effort expectations, and provide users with a pleasant and comfortable riding experience and easy installation and use, user approval will increase. It is believed that in developing countries where child safety seat legislation is not perfect, the design and customization of child safety seats to meet the demands of users would benefit more children.

Table 2 shows that 63.72% of respondents are willing to spend less than 4000 yuan to buy a smart seat, while the current price of the smart seat is between 4699 yuan and 7999 yuan, which means that most people may feel that the smart seat is too expensive and are unwilling to buy it.

At present, the reason for the high cost of our child safety seat is that the sales volume is too low. Because the cost of mold, material, and marketing is spread equally across each safety seat, which is very expensive, if it sells around 300,000 units a year, the cost could be significantly reduced, bringing it down to the same price as a conventional safety seat on the market.

## 5. Conclusions

Currently, many studies show a high rate of people abusing child restraints [50,51,52,53,54,55,56] (such as improper installation, and improper lap belt position [57].) International child safety seat survey statistics show that two thirds of car seats are incorrectly fitted. In order to increase appropriate use of car seats, the Isofix- innovative system was created. The aim of Isofix is to help secure the seat into the car and create a much simpler and more secure installation [58]. Ahmad, Y et al. found that compared with three-point seat belts, the combination of Isofix and Top Tether provided better safety performance in reducing injuries to child occupants. The Isofix system improved CRS performance in dummies aged three years and 18 months. For three-year-old dummies, head and chest injuries improved by 24% and 14%, respectively [59].

This research aim is to further facilitate Isofix, adding innovative functionality- tightening Isofix automatically [60,61,62]. We believe this new feature could enhance child safety in vehicles. Discomfort and heating can also be minimized as part of these technical enhancements and new additions to the car design. Overall design improvements no doubt would increase appropriate child safety seat use.

However, our experience shows that it is not enough to complete the stage from the laboratory to commercialization. The development of user attitudes and behavior needs to be closely monitored to increase user purchases and usage. In order to better understand and successfully promote child restraint, it is necessary to use our research method [28] to analyze user behavior, which means that emphasizing convenience, hedonic motivation, performance expectation, effort expectancy and improving social influence in marketing is a necessary condition to increase users’ willingness to use the seat.

First, in order to let more users know about intelligent safety seats, free experience activities can be provided to customers at dealers to demonstrate the convenience and compatibility of intelligent safety seat installation and dispel peoples’ concerns. Secondly, intelligent safety seats can be installed in taxis so that children can use them free of charge and experience their convenience, safety, and comfort. Third-party online and offline social networks can also play a more important role in promoting smart safety seats, as friends, family, and colleagues represent important and trusted information sources. Marketers can design “bring a friend for free” events to harness the potential of trusted social connections. Fourth, government agencies could team up with private groups (like car clubs and early education classes) to promote the benefits of smart child restraints through joint education campaigns.

## 6. Limitations and Future Research

The limitation of this research are, however, that design improvements could only be applicable to cars built with Isofix or fairly newer vehicles.

**Figure 14:**
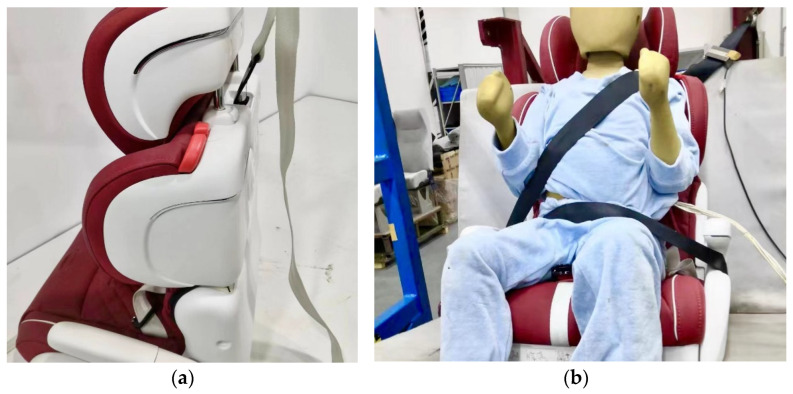
(**a**) Intelligent child restraints can be attached using the flexible Latch belt; (**b**) Intelligent child restraints can be attached using the vehicle seat belt.

For vehicles without Isofix, our child restraints are attached using the flexible Latch belt and the vehicle seat belt. Please refer to Figure 14.

However, such a connection is unstable, and the child safety seat will shift during the driving process. We will further investigate the use of flexible connections or the addition of support legs to stabilize child safety seats in vehicles without Isofix.

Since the intelligently modified child safety seats are only appropriate for children aged three to six, the next step will be to design booster seats for older children. Since the sensors, heating, ventilation, and Isofix electric devices of this safety seat are all located in the seat cushion, the heating and ventilation functions, as well as the alarm function for leaving children unattended in a vehicle, will be retained in the new booster seats.

We will introduce a new model that replaces the leather cover with a cheaper cloth cover to make intelligent child restraint more accessible to families with lower incomes. Alternatively, advocate for their purchase by governments or charities to provide them at no cost to children from low-income families.

Future research may extend this intelligent transformation to other age groups of child safety seats and gradually use technology to lower the price. These products will likely encourage more children in developing nations to use child safety seats and thereby prevent injuries in the event of road accidents.

## 7. Patents

The safety seat studied in this paper has been granted the following patents:

(1) Lin; Hao. Electrical foldable car seat [P]. US 20180111515 A1, 2018-04-26
(2) Lin; Hao. Foldable car seat [P]. US 20180111516 A1, 2018-04-26
(3) Lin; Hao. Child-safety chair [P]. US D816356 S, 2018-05-01
(4) Lin; Hao. Adjustment system and method thereof between a child car safety seat and a backrest [P]. US 20190160980 A1, 2019-05-30
(5) Lin; Hao. System and method for linkage adjustment of Isofix and backrest of child safety seat [P]. EP3444144, 2018-07-12
(6) Lin; Hao. Folding electric child safety seat [P]. EP3248835, 2017-10-05

## Figures and Tables

**Table 1:** Demographic characteristics of respondents (N = 1020).

Demographic Profile	Number	Percentage
Gender		
Female	389	38.1
Male	631	61.9
Age (years)		
≤25	187	18.3
26–35	582	57.1
36–45	214	21.0
>45	37	3.6
Education level		
10–12 years	93	9.1
College degree	186	18.2
Bachelor	645	63.2
Post-graduate degree/PhD	96	9.4
Annual household income (RMB)		
≤100,000 Yuan	176	17.3
100,000–300,000 Yuan	473	46.4
300,000–500,000 Yuan	243	23.8
>500,000 Yuan	128	12.5
Travel times (per month)		
0–10 times	640	62.7
11–20 times	285	27.9
21–30 times	95	9.4

**Table 2:** Respondents’ acceptance of child safety seats was compared.

Question	M	SD	*n*	Response Categories						
2022				Completely Disagree (1)	Disagree (2)	Partially Disagree (3)	Neutral (4)	Partially Agree (5)	Agree (6)	Completely Agree (7)
IU1: If I can afford it, I would like to buy a smart child restraint.	4.89	1.14	1020	0.00%	0.49%	8.82%	28.82%	36.96%	12.75%	12.16%
IU2: I intend to use the smart child restraint extensively in the future.	4.85	1.16	1020	0.00%	1.18%	9.22%	29.41%	35.98%	12.25%	11.96%
IU3: I’d want to try the smart child restraint.	4.75	1.11	1020	0.00%	0.88%	10.10%	32.25%	35.59%	11.96%	9.22%
2016										
I want to use this child restraint.	3.83	1.25	392	1.53%	11.22%	30.10%	28.83%	18.62%	7.14%	2.55%

Abbreviations: M = means; SD = standard deviations; *n* = number of respondents.

**Table 3:** The price respondents are willing to pay.

Question	*n*	Under 3000 RMB	3001~4000 RMB	4001~5000 RMB	5001~6000 RMB	Over 6000 RMB
How much are you willing to pay for a smart child safety seat?	1020	31.96%	31.76%	22.84%	9.12%	4.31%

Abbreviations: n = number of respondents.

**Table 4:** The outcomes of a confirmative factor analysis.

Latent Variable	Observed Variable	ƛ	Cronbach’s α	AVE	CR
Performance Expectancy			0.896	0.686	0.897
	PE1	0.865			
	PE2	0.813			
	PE3	0.846			
	PE4	0.789			
Effort Expectancy			0.874	0.698	0.874
	EE1	0.831			
	EE2	0.837			
	EE3	0.838			
Social impact			0.860	0.670	0.859
	SI1	0.830			
	SI2	0.814			
	SI3	0.812			
Convenient conditions			0.860	0.673	0.860
	CC1	0.834			
	CC2	0.818			
	CC3	0.809			
Hedonic incentive			0.877	0.704	0.877
	HI1	0.832			
	HI2	0.838			
	HI3	0.847			
Intention to use			0.872	0.695	0.872
	IU1	0.856			
	IU2	0.822			
	IU3	0.822			

Note: The measurement of the UTAUT2 components was adapted to the setting of this study based on [22]. ƛ = factor loading; α = Cronbach’s alpha; CR = construct reliability; AVE = average variance extracted, a measure of convergence among observable variables reflecting a latent variable [42].

**Table 5:** KMO value and Bartlett’s sphericity test.

KMO	Bartlett Spherical Test		
	The approximate chi-square	d.f.	*p*
0.984	17,825.667	171	0.000 ***

Abbreviations: KMO = Kaiser-Mayer-Olkin. Note: *** denote 1% significance levels.

**Table 6:** Latent variable correlation.

	AVE	BI	HM	FC	EE	PE	SI
IU	0.695	0.834					
HI	0.704	0.577	0.839				
CC	0.673	0.534	0.484	0.820			
EE	0.698	0.463	0.404	0.468	0.835		
PE	0.686	0.520	0.503	0.487	0.452	0.828	
SI	0.670	0.555	0.59 7	0.476	0.437	0.497	0.819

Abbreviations: IU = Intention to use; HI = Hedonic incentive; CC = Convenient conditions; EE = Effort expectancy; PE = Performance expectancy; and SI = Social impact.

**Table 7:** Model Fit Measures (Recommended Values).

Fit Indices	χ^2^/df	GFI	RMSEA	RMR	CFI	NFI	NNFI
value	3.469	0.974	0.049	0.023	0.981	0.974	0.976
Cut-off criterion	<5	>0.9	<0.10	<0.05	>0.9	>0.9	>0.9

Abbreviations: χ^2^/df = ratio of chi-square to degrees of freedom; GFI = goodness-of-fit index; RMSEA = root mean square error of approximation; RMR = root mean square residual; CFI = comparative fit index; NFI = normed fit index; and NNFI = non normed fit index.

**Table 8:** Results of structural equation modeling.

Hypotheses	Regressive Path			Coef.	Std. Estimate	S.E.	C.R.	*p*	Hypothesis Validation
H1	PE	→	IU	0.223	0.344	0.019	11.687	***	Supported
H2	EE	→	IU	0.089	0.146	0.017	5.341	***	Supported
H3	SI	→	IU	0.131	0.233	0.016	8.234	***	Supported
H4	CC	→	IU	0.419	0.677	0.023	18.246	***	Supported
H5	HI	→	IU	0.283	0.497	0.018	15.67	***	Supported

Abbreviations: Coef. = un-standard estimated coefficients; Std. Estimate = standard estimated coefficient; S.E.= Std. Error; C.R. = dividing the regression weight estimate by the estimate of its standard error gives. Significance of Correlations: *** = *p* < 0.001.

**Table 9:** Model Comparison.

Model	DF	CMIN	*p*	∆TLI	∆CFI
Nested Model Comparisons for gender					
Measurement weights	13	15.355	0.286	−0.017	0
Structural weights	5	12.763	0.026 *	−0.005	0
Structural covariances	5	3.107	0.683	−0.006	0
Nested Model Comparisons for age					
Measurement weights	13	13.245	0.429	−0.006	0
Structural weights	5	6.111	0.296	−0.002	0
Structural covariances	5	6.533	0.258	−0.002	0
Nested Model Comparisons for education					
Measurement weights	13	9.874	0.704	−0.006	0
Structural weights	5	21.715	0.001 *	−0.001	−0.001
Structural covariances	5	5.071	0.407	−0.002	0
Nested Model Comparisons for travel times					
Measurement weights	13	26.856	0.013	0.017	−0.001
Structural weights	5	8.485	0.131	−0.005	0
Structural covariances	5	1.616	0.899	−0.006	0

* *p* < 0.05.

**Figure A1:**
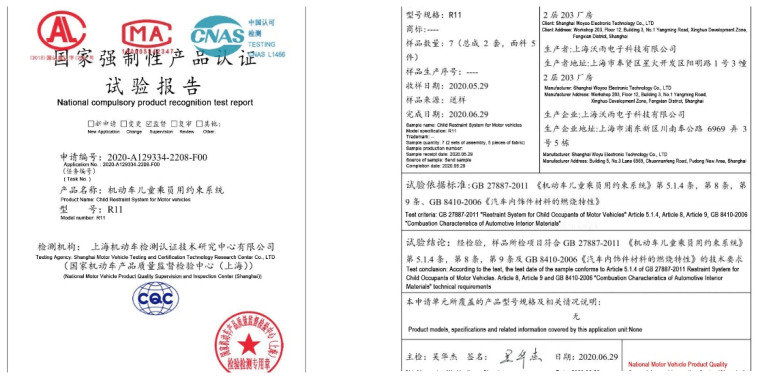
The intelligent child restraint system studied in this paper has passed China’s national compulsory product certification test (including crash test).

## Data Availability

Not applicable.

## Appendix A

**Table A1:** Measuring Instrument.

Questionnaire Construction	Derived From
Performance expectancy	[32]
PE1: I think the child restraint would be good for my driving.	The system would be helpful in my line of work.
PE2: I could go to my destination more safely if I used the child restraint.	
PE3: The child restraint would make it easier for me to go where I’m going.	There is a particular individual (or group) who can help with system issues.
PE4: I could get to my destination faster if I used the child restraint.	
Effort expectancy	[32]
EE1: I have no trouble picking up how to use the child restraint.	The system would be simple for me to utilize.
EE2: I think the system is simple to use.	My ability to learn how to use the technology is excellent.
EE3: I would communicate clearly and simply using the child restraint.	I would have a clear and understandable relationship with the system.
Social impact	[32]
SI1: I should use child restraint, according to people that matter to me.	My closest friends and relatives advise me to use the system.
SI2: Those who have the power to affect my behavior believe I should employ child restraint.	I should use the system, according to those who have power over my behavior.
SI3: People whose viewpoints I respect want me to use child restraint.	
Convenient conditions	[32]
CC1: I am equipped with the resources required to use the child restraint.	I have everything I need to use the system.
CC2: I am knowledgeable enough to use the child restraint.	I am knowledgeable enough to operate the system.
CC3: The child restraint would be compatible with the car and phone I use	My previous systems and this one are incompatible.
Hedonic incentive	[22]
HI1: Using the child restraint would be fun during car trips	Mobile Internet use is enjoyable.
HI2: Using the child restraint would be enjoyable	It’s fun using mobile Internet.
HI3: Using the child restraint would make me and my kids very happy during car trips	Using the mobile Internet is a lot of fun.
Intention to use	[22]
IU1: If I can afford it, I would like to buy an child restraint	I’ll make an effort to use the mobile Internet as much as possible.
IU2: I intend to use child restraint extensively in the future.	I’m going to keep using mobile Internet a lot.
IU3: I’d want to try the child restraint.	In the future, I’m going to keep using mobile Internet.

## Appendix B

This smart child safety seat has passed the safety test of the National Motor Vehicle Product Quality Supervision and Inspection Center and meets the technical requirements of GB 27887-2011 “Restraint System for Child Occupants of Motor Vehicles” Article 5.1.4, Article 8, Article 9 and GB8410-2006 “Combustion Characteristics of Automotive Interior Trim Materials.” Report number: 05601-JD20L1204.
